# Chemopreventive Effects of Polysaccharides and Flavonoids from Okra Flowers in Azomethane/Dextran Sulfate Sodium-Induced Murine Colitis-Associated Cancer

**DOI:** 10.3390/nu15224820

**Published:** 2023-11-17

**Authors:** Yuanle Deng, Xiaoyi Huang, Xiaotong Chen, Meng Wang, Li Tian, Heting Zhou, Wenyu Yang, Fang He, Wenya Yin

**Affiliations:** 1 West China School of Public Health and West China Fourth Hospital, Sichuan University, Chengdu 610041, China; xiaole1112@163.com (Y.D.);; 2 Department of Clinical Nutrition, Sichuan Clinical Research Center for Cancer, Sichuan Cancer Hospital & Institute, Sichuan Cancer Center, Affiliated Cancer Hospital of University of Electronic Science and Technology of China, Chengdu 610041, China; 3 Department of Clinical Nutrition, Sichuan Provincial People’s Hospital, University of Electronic Science and Technology of China, Chengdu 610072, China; 4 Pharmaceutical Engineering, School of Food Science and Bioengineering, Xihua University, Chengdu 610039, China

**Keywords:** okra flowers, polysaccharide, flavonoids, colitis-associated cancer, inflammation

## Abstract

Okra flowers are a good source of polysaccharides and flavonoids, with biological activities of anti-inflammatory action and modulation of the gut microbiota. Previously, we reported that flavonoid-rich extracts from okra flowers (AFE) presented effective anti-colorectal cancer (CRC) activity in CRC cells as well as xenograft models, but their role in colitis-associated cancer (CAC) is unidentified. In this study, we aimed to evaluate the effects of AFE and APE (polysaccharides extracted from okra flowers) on the CAC symptoms of azoxymethane (AOM)/dextran sodium sulfate (DSS)-intervened mice. The results showed that APE and AFE exert potent efficacy in inhibiting colitis and colorectal tumorigenesis stimulated by AOM/DSS, characterized by decreased colonic shortening, DAI score, and tumor numbers. Compared with the control group, APE/AFE alleviated the microbiota dysbiosis driven by AOM/DSS. In addition, AFE elicited its anticancer activity through regulation of NFκB/IL-6/Stat3, JAK2/Stat3, MAPKs, PI3K/AKT, and Wnt/β-catenin signal transductions in AOM/DSS mice, which was consistent with a vitro model of CT26 cells, while APE treatment exhibited anticancer activity through regulation of Nrf2/IL-6, MAPKs, PI3K/AKT, and Wnt/β-catenin signal transductions in the AOM/DSS mouse model. Collectively, our studies revealed, for the first time, that flavonoids and polysaccharides from okra flowers possess the ability to attenuate colitis and colorectal tumorigenesis, with them having great potential to become promising candidates against CRC.

## 1. Introduction

Colorectal cancer (CRC) remains a principal cancer type and the primary cause of death worldwide, although substantial progress has been achieved in CRC therapy over recent years. According to the GLOBOCAN 2020 estimate, CRC ranks third (more than 1.9 million new CRC cases) in morbidity and second (935,000 deaths) in mortality among various malignancies [1]. Stemming from focal or multifocal patchy and flat dysplasia in inflammatory regions, colitis-associated cancer (CAC) is a fatal complication of inflammatory bowel disease (IBD) and the most common type of CRC [2]. IBD can significantly increase the risk of developing CRC and is correlated positively with the degree, range, and length of disease [3]; for example, a longer course of IBD generally provides more risk for developing CRC [4]. Of note, as a critical potential pathogeny of colorectal tumorigenesis, chronic inflammation can exist from the beginning phase of tumorigenesis [5,6] and facilitate the development of CRC [7].

Nowadays, the relevance between phytochemicals and CRC has been reported in epidemiological studies as well as experimental studies. Many epidemiological studies have emphasized the correlation between the intake of several nutrients or foods enriched in phytochemicals and lower risk of CRC [8] and have spotted the chemopreventive activities of natural compounds in preventing or reversing the process of carcinogenesis [9]. In addition, experimental studies have revealed that dietary phytochemicals can exhibit chemopreventive effects against CRC through adjusting related signaling pathways or modulating the gut microbiota [8]. 

Okra (*Abelmoschus esculentus* L.), a flowering plant of Malvaceae, is extensively planted worldwide and is usually eaten as a vegetable. Okra flowers are a byproduct of okra and are partially consumed as healthy tea, while the vast majority are discarded as a waste of resources [10]. Surprisingly, okra flowers are enriched in flavonoids [11] and polysaccharides [12], two important bioactive compounds in plants, which have the potential to be novel agents or food resources for modulating intestinal health and controlling intestinal disease. Our previous study found that AFE (*Abelmoschus esculentus* (Linn.) Moench flower flavonoid extract) extracted from okra flowers markedly inhibited the growth of CRC cells through the induction of mitochondrial dysfunction-associated apoptosis, senescence, and autophagy, without adverse influence on the cytoactivity of normal cells [13]. And in vivo, AFE showed significant antitumor effects on tumor xenograft model mice without apparent toxicity and side effects; the preventive action of AFE was more visible than its curative action [13]. Neither cell models nor xenograft mouse models can fully reflect the growth process of human cancer as well as the characteristics and effectiveness of agents in the clinic. And azoxymethane (AOM) combined with dextran sodium sulfate (DSS) has mostly been applied to induce the CAC mouse model, which mimics the colonic inflammation and carcinogenesis circumstances in humans and has similarities to the features and processes of human CRC; thus, it is superior in reflecting the features and effects of antitumor agents in clinical applications [14]. Moreover, chronic intestinal inflammation affects the progression of CAC by facilitating inflammatory signaling pathways and cell proliferation and changes in immune response, thus facilitating tumorigenesis [15,16]. Experimental models of CAC may help clarify how chronic inflammation and related signaling pathways exert action in the initiation and progression of CRC. 

The flavonoids and polysaccharides from okra flowers possess the features of high obtainment rates, safety, and low economic costs, but there is no record of them in the CAC literature. Thus, the objective of our study was to identify the role of okra flower-derived polysaccharides and flavonoids in alleviating colitis and CAC tumorigenesis in AOM/DSS-induced CAC mice and to elucidate the influencing pathway regulated by APE (*Abelmoschus esculentus* (Linn.) Moench flower polysaccharide extract) and AFE. We applied the flavonoids and polysaccharides from okra flowers to chemically induced the CRC mouse model first, and collectively, the current study might offer a scientific basis for applying okra flower-derived polysaccharides and flavonoids as novel promising candidates in controlling CRC.

## 2. Materials and Methods

### 2.1. Preparation of AFE and APE

The method of APE preparation is illustrated in Figure S2A and described as below. After extraction twice with 75% ethanol under reflux for 2 h (about 70 °C), the filtrates were filtered and purified to acquire AFE, and the residue was then extracted twice with double-distilled water (ddH_2_O) at 70 °C for 3 h. After filtering to throw off the residual and concentrating the extracts under vacuum at 70 °C, anhydrous ethanol was used to precipitate the polysaccharide extracts (the ratio of concentrated solution to ethanol was 1:3), and they were kept overnight for half a day at 4 °C. The precipitate was collected and then dissolved in ddH_2_O. The solution was precipitated again by anhydrous ethanol to an eventual ethanol dose of 75% and kept overnight for half a day at 4 °C. After being washed with anhydrous ethanol, the precipitate fraction was resolved in ddH_2_O and dried into lyophilized powder through vacuum freezing. Additionally, AFE was extracted and purified from okra flowers, according to our previous study [13], sequentially: 70% ethanol extraction (70 °C) of okra flowers, purification via AB-8 macroporous resin, and freeze-drying of the eluent. 

### 2.2. Cell Lines and Cell Culture

CT26 (ATCC, Rockville, MD, USA), murine CRC cells were cultivated in RPMI 1640 medium with 10% heat-inactivated fetal bovine serum (FBS) and 1% antibiotics (penicillin and streptomycin) added. The cells were cultivated in a humidified atmosphere of 5% CO_2_ at 37 °C. The cells were exposed to the designated concentrations of AFE (0, 50, 100, and 200 µg/mL) for a whole day at 37 °C after being attached to Petri dishes.

### 2.3. Mice and Animal Experiments 

#### 2.3.1. Mice

C57BL/6 mice (male; weighing 18–20 g) at six weeks of age from Beijing Vital River Laboratory Animal Technology Co., Ltd. (Beijing, China) were cultured in a specific pathogen-free (SPF) environment (temperature controlled: 22–29 °C) and received their food and drink freely. 

Our animal experiment was permitted by the Medical Ethics Committee of Sichuan University in China (new permit number: Gwll2021058).

#### 2.3.2. AOM/DSS Animal Study

**Figure 1:**
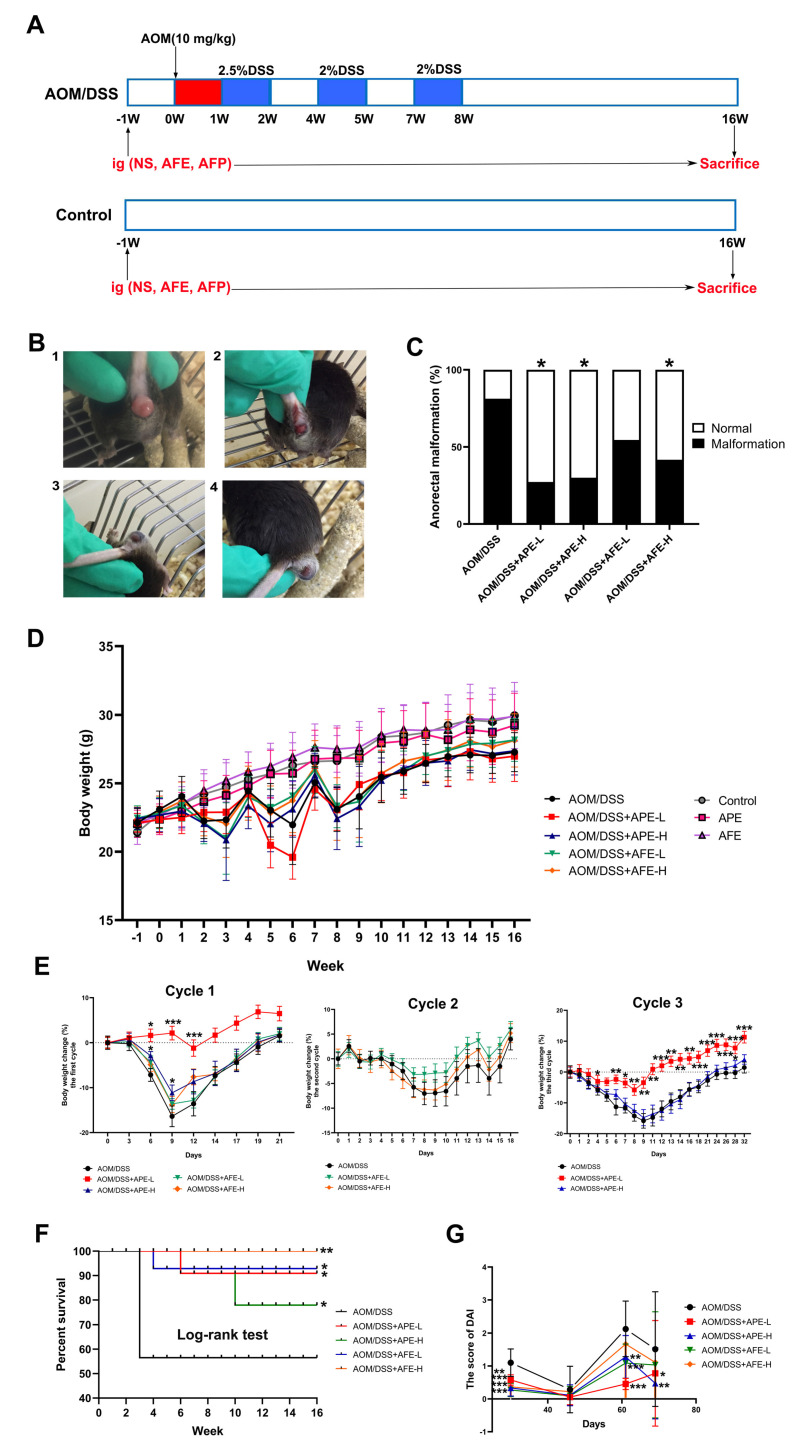
Effects of APE and AFE on the physiological indices of the CAC mice. (**A**) Schematic diagram of the animal experimental study. (**B**) Representative appearance of the CAC mice by the end of th trial (1: anorectal prolapse; 2: anal bleeding; 3 and 4: anus turgescence and extrudes out). (**C**) The incidence rate of anorectal malformation in each group. (**D**) Changes in the body weight of the mice were recorded each week during the entire study. (**E**) Body weight changes of CAC mice in the AOM/DSS-induced groups during the three DSS cycles. (**F**) The survival curve. (**G**) The DAI score of the mice in each group. Data are presented as the mean ± SD; * *p* < 0.05, ** *p* < 0.01, *** *p* < 0.001 vs. AOM/DSS group.

The scheme of the animal experiments and design is described in Figure 1A.

After acclimation for 1 week, the mice were weighed and randomly allocated to eight groups: (1) the model group (AOM/DSS, n = 15): AOM/DSS treatment and intragastric administration of saline; (2) the APE low-dose group (APE-L, n = 12): AOM/DSS treatment and 150 mg/kg APE; (3) the APE high-dose group (APE-H, n = 12): AOM/DSS treatment and 300 mg/kg APE; (4) the AFE low-dose group (AFE-L, n = 12): AOM/DSS treatment and 150 mg/kg AFE; (5) the AFE high-dose group (AFE-H, n = 12): AOM/DSS treatment and 300 mg/kg AFE; (6) the control group (n = 10): saline; (7) APE (n = 10): 300 mg/kg APE; and (8) AFE (n = 10): 300 mg/kg AFE. 

The mouse colitis-associated cancer study was carried out based on the previous literature with mild modifications [17,18,19]. Briefly, the AOM/DSS-induced mice in the present study were intraperitoneally injected with AOM (10 mg/kg). After one week, the mice were supplied with DSS water (2.5% DSS) for 1 week (first DSS cycle) and provided with normal drinking water for the following 2 weeks. The above cycle was repeated twice with 2% DSS, and the mice were sacrificed during week 16. Both AFE and APE were administered to the mice orally once a day, starting from 1 week before the experiment (Figure 1A), while the mice in the model group and the control group received equivalent saline.

The CRC-inducing agents AOM (Sigma-Aldrich) and DSS (MW: 36,000–50,000 Da) were bought from Sigma-Aldrich and MP Biomedicals (Santa Ana, CA, USA), respectively.

#### 2.3.3. Disease Activity Index

A clinical evaluation of each mouse was executed by using an established DSS colitis disease activity index (DAI) to evaluate severity [20]. The DAI comprised the average values assigned to stool consistency, rectal bleeding, and weight loss that were logged during the experimental period (Table 1).

DAI = (Weight Loss + Stool Consistency + Fecal Bleeding)

#### 2.3.4. Enumeration of Lesions and Measurement of Colon Length

After 16 weeks, the mice were sacrificed, and the tissue from their colons was detached from the ileocecal junction to the edge of the anus swiftly. After exact measurement of length, the colon was opened longitudinally, with the adherent tissues being rid of, and then it was rinsed thrice with normal saline. The position and the number of tumors were logged. Part of the colon tissue was cut off and fixed in paraformaldehyde solution (4%) and embedded in paraffin for further pathological study. The residual tissues were placed on ice and stored at −80 °C for other experiments. 

### 2.4. Gut Microbiota Analyses

In the final stage of the experiment, fecal samples from each mouse were collected. Every mouse was individually put into the metabolic cages, and each mouse’s feces was obtained and immediately stored at −80 °C. Fecal samples from five mice from every group were applied for 16S rRNA gene sequencing. The total genomic DNA of intestinal flora from the fecal samples was extracted through the TIANamp Stool DNA Kit (DP328) and stored at −80 °C. When we finished testing the concentration and quality of the DNA through agarose gel electrophoresis, the extractive DNA was diluted to 1 ng/μL as the PCR template. And PCR amplification was carried out through universal primers 515F and 806R and specific primer barcodes. 

After removing the barcode and primer sequence, the raw tags were acquired through FLASH (V1.2.7) and QIIME (Version 1.9.1); a quality control process was carried out to gain clean tags. Ultimately, the UCHIME algorithm was applied to catch chimera sequences of the clean tags in comparison with the reference, and subsequently, the chimera sequences were abandoned to gain effective tags. Based on sequence analysis using Uparse software (Version 7.0.1001), sequences with similarity above 97% were placed into the same operational taxonomic units (OTUs). Typical sequences of every OTU were screened for subsequent annotation, and the Silva database was the reference database to used annotate the sequences. Alpha diversity indices, such as ACE, Chao1, observed_species, and PD_whole_tree were computed through QIIME (Version 1.7.0) and displayed with R software (Version 2.15.3). 

### 2.5. Hematoxylin and Eosin (H&E) Staining and Immunohistochemistry

Organs and tissues (colon and small intestine) from the mice were embedded in paraffin blocks. And sliced sections, of around 4 μm, were deparaffinized and rehydrated through a xylene–ethanol–water gradient system. The process of H&E staining was carried out according to the dehydrating process. 

In addition, immunohistochemical staining of COX2, EMR1, Ki-67, ZO-1, and occludin was performed on sections obtained from the paraffin-embedded small intestine and colon. After deparaffinization in citrate buffer solution, the sections were preincubated for 25 min in H_2_O_2_ solution (3%) and then re-incubated for 1 h in regular goat serum. The sections were incubated with primary antibodies in a refrigerator (4 °C) overnight. And the slides were incubated in HRP-conjugated secondary antibody solution for around 50 min. Then, the sections were covered with DAB for minutes and counterstained for 3 min with hematoxylin. Eventually, the sections were dehydrated with ethanol, sealed, and analyzed through image analysis software, and every section stained was watched and photographed. ImageJ software (V1.8.0) was used to quantify the IHC data.

### 2.6. Western Blot Analysis

The protein from the CT26 cells and the tissue from the colon collected from the AOM/DSS-treated mice were lysed in frozen RIPA buffer solution (the cocktails (protease inhibitor and phosphatase inhibitor) were added). The supernatant of the solution was obtained through centrifugation (13,300 rpm, 15 min) in a refrigerator (4 °C). Furthermore, the BCA Protein Assay Kit (Beyotime Biotech, Beijing, China) was applied to detect the concentration of protein, and the samples (30–50 μg) were loaded onto SDS-PAGE gel for electrophoresis. And the protein from the samples was electrotransferred onto the PVDF membranes after gel electrophoresis. The membranes were blocked in Tris-buffered saline solution comprising 5% skimmed milk and 1% Tween 20 (TBST, pH 7.4) for around 1 h and incubated with the indicated antibody overnight. Following washing with TBST five times, the membranes were incubated with the relevant HRP-conjugated secondary antibody (1:2000 dilution) at room temperature for 2 h and then washed with TBST another five times. Finally, the blots were developed with an enhanced chemiluminescence kit, and the monoclonal GAPDH antibody was used as the internal control.

### 2.7. Statistical Analysis

The results are expressed as the mean ± standard deviation (SD). One-way ANOVA followed by Dunnett’s *t*-test in SPSS 16.0 software was used to examine the differences between the groups. GraphPad Prism software (version 7.0) was used to generate the figures in our data. The survival analysis was carried out through the Kaplan–Meier method (log-rank test). And the following *p*-values were considered statistically significant: * *p* < 0.05; ** *p* < 0.01; *** *p* < 0.001.

## 3. Results

### 3.1. APE and AFE Attenuated the AOM/DSS-Induced Physiological Index

Firstly, we tested the ability of APE and AFE to relieve AOM/DSS-induced CAC, which was developed by AOM and three cycles of DSS (Figure 1A). At the end of the experiment, typical anorectal features of the mice, including redness, swelling, bloodshed, and anorectal prolapse, which are successful indicators of the AOM/DSS-induced CAC model, were observed (Figure 1B). The incidence rate of anorectal malformation in each group was recorded, and it was observed that APE and AFE attenuated the occurrence of anorectal abnormalities (*p* < 0.05) in comparison with the model group (Figure 1C). Moreover, the body weight of the mice was recorded weekly throughout the experiment (Figure 1D), and compared to the control group, the body weight of the CAC mice decreased obviously, but it reversed after the subsequent administration of normal drinking water in the three DSS cycles. The weight loss of the AOM/DSS group reached the highest point on the nineth day after the CAC mice drank the DSS solution freely. And the mice in the APE and AFE intervention groups lost less body weight after DSS treatment and recovered faster than the mice in the AOM/DSS group (Figure 1E). 

Furthermore, survival rate is also a significant index to evaluate the therapeutic effect of agents on CRC treatment, and the death rate of all groups was monitored weekly. Clearly, the survival rate of the AOM/DSS group was the lowest among all groups, while both APE and AFE administration elevated the survival rate of the AOM/DSS-induced CRC mice (Figure 1F). Like human IBD, the AOM/DSS-induced model mice exhibited apparent clinical features of chronic colitis, including body weight loss, diarrhea, and bloody stools, resulting in markedly increased DAI scores. Compared to the AOM/DSS group, less weight loss and more slight diarrhea and bloody stools were observed in the APE and AFE-treated groups, and therefore, the their DAI score was significantly reduced (Figure 1G). These results suggest that APE and AFE can effectively alleviate colitis signs in CAC mice. 

### 3.2. APE and AFE Attenuate AOM/DSS-Induced Colorectal Carcinogensis

**Figure 2:**
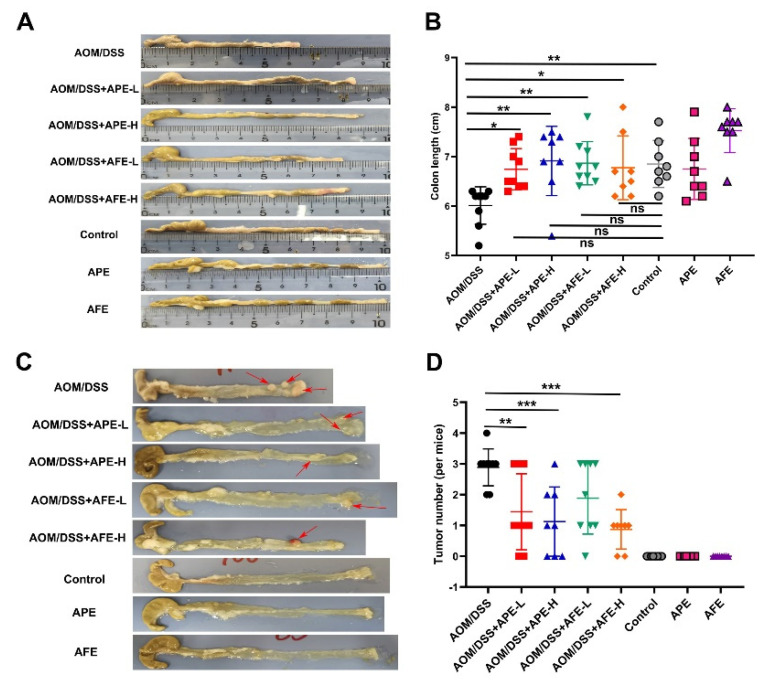
Inhibitory activities of APE and AFE in AOM/DSS-induced colitis-associated tumorigenesis. (**A**) Representative photographs of colonic tissues. (**B**) Length of the colons in the different groups. (**C**) Typical photographs of longitudinal anatomical colons (red arrow: tumor). (**D**) Number of colon polyps in different groups. Data are presented as the mean ± SD; * *p* < 0.05, ** *p* < 0.01, *** *p* < 0.001 vs. AOM/DSS group.

Increased inflammation of the colon is always positively associated with a decrease in colon length, in the present study, and the colon length of the model group was significantly shorter than the control group, while the intervention with APE and AFE markedly enhanced the colon length of the AOM/DSS-treated mice (Figure 2A,B). Upon necropsy, the CAC mice displayed bunchy neoplasms near their anus in the middle and posterior segments of their colon, and every mouse in the groups that were exposed to AOM/DSS developed tumors. But compared to the model group, there were substantially fewer total numbers of tumors in the APE and AFE administration groups (Figure 2C,D).

### 3.3. APE and AFE Protect Organs against AOM/DSS-Induced Injury

**Figure 3:**
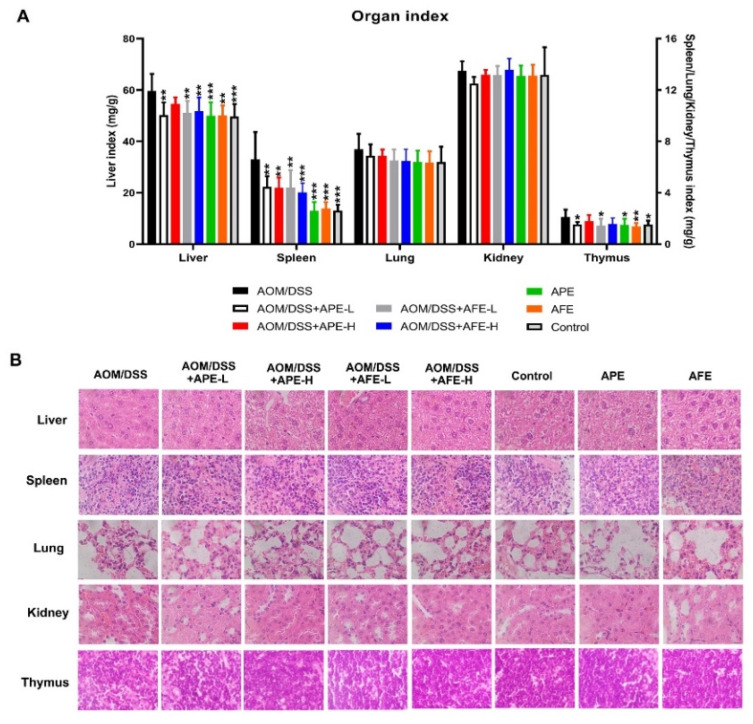
Toxicity evaluation of APE and AFE. (**A**) The organ coefficients (liver, spleen, lungs, kidneys, and thymus) of each group. (**B**) H&E staining of organs (liver, spleen, lungs, kidneys, and thymus). Data are presented as the mean ± SD; * *p* < 0.05, ** *p* < 0.01, *** *p* < 0.001 vs. AOM/DSS group.

The results in Figure 3A show that the CAC mice exhibited a more significant increase in the liver, spleen, and thymus indices than the mice in the control group. The administration of APE and AFE significantly decreased the liver, spleen, and thymus indices induced by AOM/DSS, while a difference in the lungs and kidneys was not observed. APE and AFE displayed notable anti-CRC properties against the AOM/DSS-induced CRC model, but no potential side effects of APE and AFE were observed. Furthermore, a microscopic examination of the major organs was conducted, and no detectable morphological changes were observed in the mice treated with APE or AFE (Figure 3B).

### 3.4. APE and AFE Protect the Small Intestine and Colon against AOM/DSS-Induced Injury

**Figure 4:**
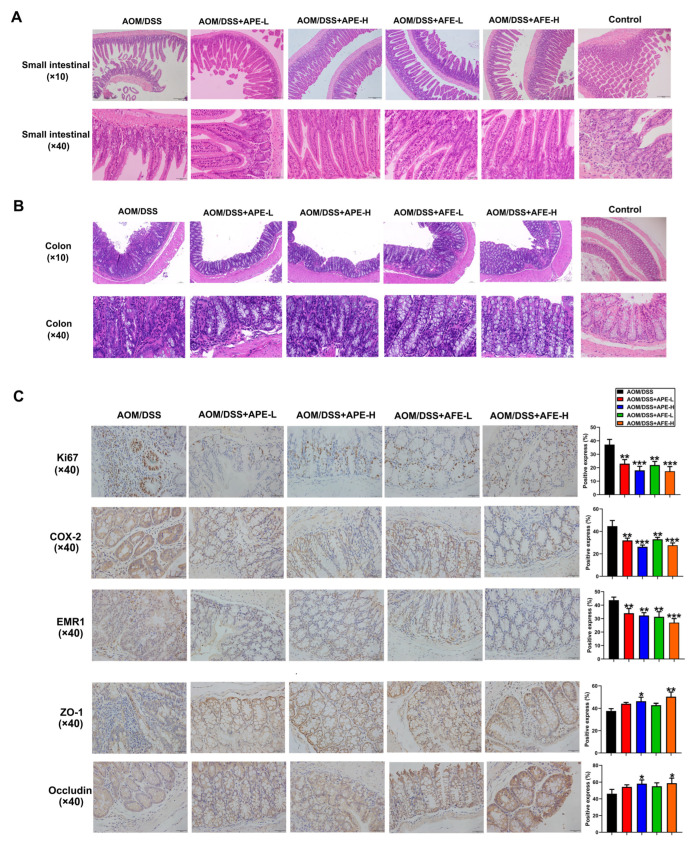
APE and AFE inhibited AOM/DSS-induced inflammation and histological injury in the small intestinal and colonic tissue. (**A**) H&E-stained pathological sections of the small intestinal tissues. Scale bar, 100 μm (upper panel) and 20 μm (lower panel). (**B**) H&E-stained pathological sections of colonic tissues. Scale bar, 100 μm (upper panel) and 20 μm (lower panel). (**C**) IHC staining of ZO-1, occludin, Ki67, COX-2, and EMR1 expression in colonic tissues. Data are presented as the mean ± SD; * *p* < 0.05, ** *p* < 0.01, *** *p* < 0.001 vs. AOM/DSS group.

Histopathological examination of the small intestine showed that, AOM/DSS treatment led to inflammatory cell infiltration, the decrease of intestinal villi, the loss of intestinal crypt architecture, and edema, while APE and AFE treatment inhibited the pathological changes (Figure 4A). Histopathological examination (Figure 4B) implied that the colon of the mice in the AOM/DSS group had developed chronic inflammation, featuring colonic mucosal ulcers, epithelium disruption with a loss of epithelial integrity and abnormal glands, and remarkable immune cell infiltration. APE and AFE significantly alleviated pathological changes, such as mucosal damage, the infiltration of inflammatory cells, and abnormal glands. 

Immunohistochemistry examination showed that CRC earl markers, including Ki67, COX2, and EMR1, were highly expressed in the colons in the model group, but these indicators showed lower expression in the APE and AFE intervention groups than in the model group. Moreover, we tested the expression of zonula occludens-1 (ZO-1) and occludin in the colon tissues, and found that, compared to the AOM/DSS group, the expression of ZO-1 and occludin was obviously elevated in the colon by APE and AFE treatment.

### 3.5. APE and AFE Inhibited Colonic Inflammation in the AOM/DSS Mice by Regulating the Inflammatory Signaling Pathway

#### 3.5.1. APE and AFE Regulated IL-6 and Related Signaling Pathways in the AOM/DSS Mice

**Figure 5:**
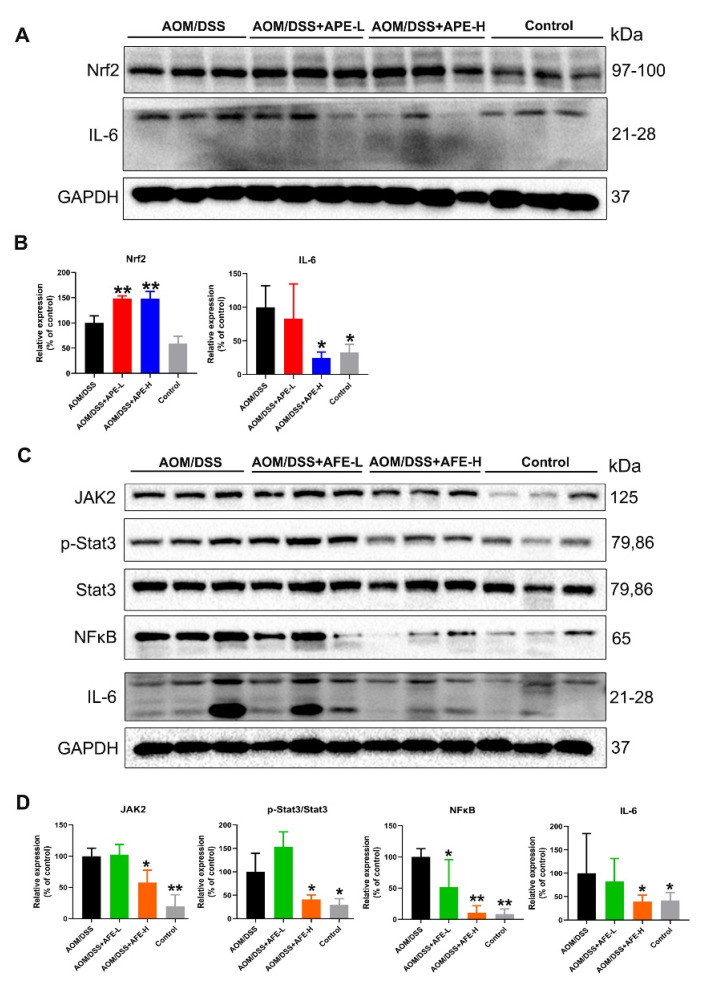
APE and AFE inhibited colonic inflammation in the CAC mice. (**A**) APE downregulated the level of Nrf2 and IL-6 in colonic tissues. (**B**) The relative intensities of Nrf2 and IL-6 expression after normalization to GAPDH. (**C**) AFE inhibited the level of JAK2, p-Stat3/Stat3, NfκB, and IL-6 in colonic tissues. (**D**) The relative intensities of JAK2, p-Stat3/Stat3, NfκB and IL-6 expression after normalization to GAPDH. Data are presented as the mean ± SD; * *p* < 0.05, ** *p* < 0.01 vs. AOM/DSS group.

The Western blotting results in Figure 5A,B show that AOM/DSS treatment decreased transcription factor nuclear factor erythroid 2-related factor 2 (Nrf2) expression and increased interleukin-6 (IL-6) expression in comparison with those of the control group, while APE treatment increased Nrf2 expression and decreased IL-6 expression, compared to the model group, which suggests that APE might downregulate the Nrf2-mediated expression of inflammatory mediators.

We also found that NFκB signaling components were elevated in the model mice, as well as the protein expression of JAK2, p-Stat3/Stat3, and IL-6 (Figure 5C,D), suggesting increased colitis in the model group. However, AFE administration markedly inhibited the expression of these NFκB signaling component molecules and JAK2 compared to the AOM/DSS mice, which indicated that AFE could alleviate AOM/DSS-induced CAC by downregulating the NFκB/IL-6/Stat3 and JAK2/Stat3 pathway.

#### 3.5.2. APE and AFE Regulated the MAPKs Signaling Pathway in the AOM/DSS Mice

**Figure 6:**
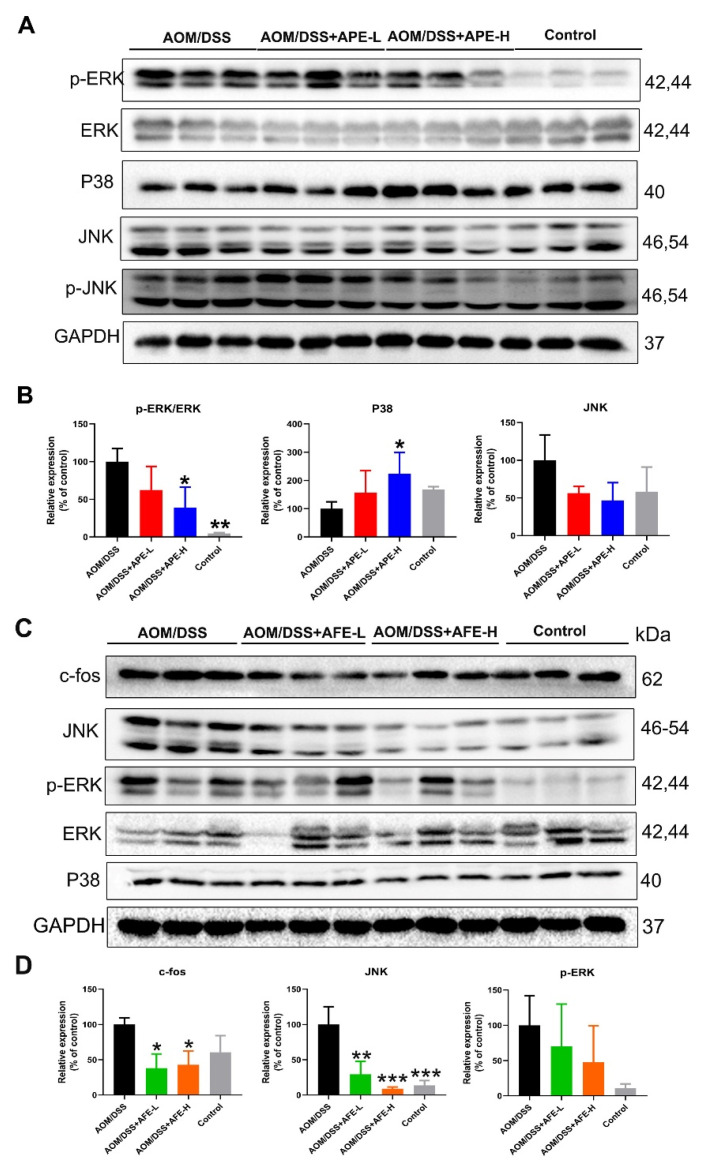
APE and AFE inhibited the MAPK signaling pathway in the colonic tissues of the CAC mice, as determined via Western blotting. (**A**) Effects of APE on p-ERK/ERK, P38, and p-JNK/JNK in the colonic tissues of the CAC mice. (**B**) The relative intensities of the expression of (**A**) normalized against total proteins. (**C**) Effects of AFE on p-ERK/ERK, P38, JNK, and c-fos in the colonic tissues of the CAC mice. (**D**) The relative intensities of the expression of (**C**). Data are presented as the mean ± SD; * *p* < 0.05, ** *p* < 0.01, *** *p* < 0.001 vs. AOM/DSS group.

We studied mitogen-activated protein kinases (MAPKs), the key efficient molecule that mediates proinflammatory signaling, and Western blot analysis showed (Figure 6A–D) that the relative levels of JNK and p-ERK were upregulated in the AOM/DSS group. While in comparison with the AOM/DSS group, APE intervention markedly suppressed the phosphorylation of ERK and activated P38 expression, and AFE administration markedly inhibited p-ERK, c-fos, and the downstream target gene c-fos. These above data suggest that APE and AFE conferred prominent protective effects against AOM/DSS-induced CAC in the C57BL/6 mice by suppressing inflammatory responses such as the regulation of the MAPKs signaling pathways. 

### 3.6. APE and AFE Inhibited the CRC-Associated Signaling Pathways

#### 3.6.1. APE and AFE Inhibited PI3K/AKT/mTOR Signal Transduction in the AOM/DSS Mice

**Figure 7:**
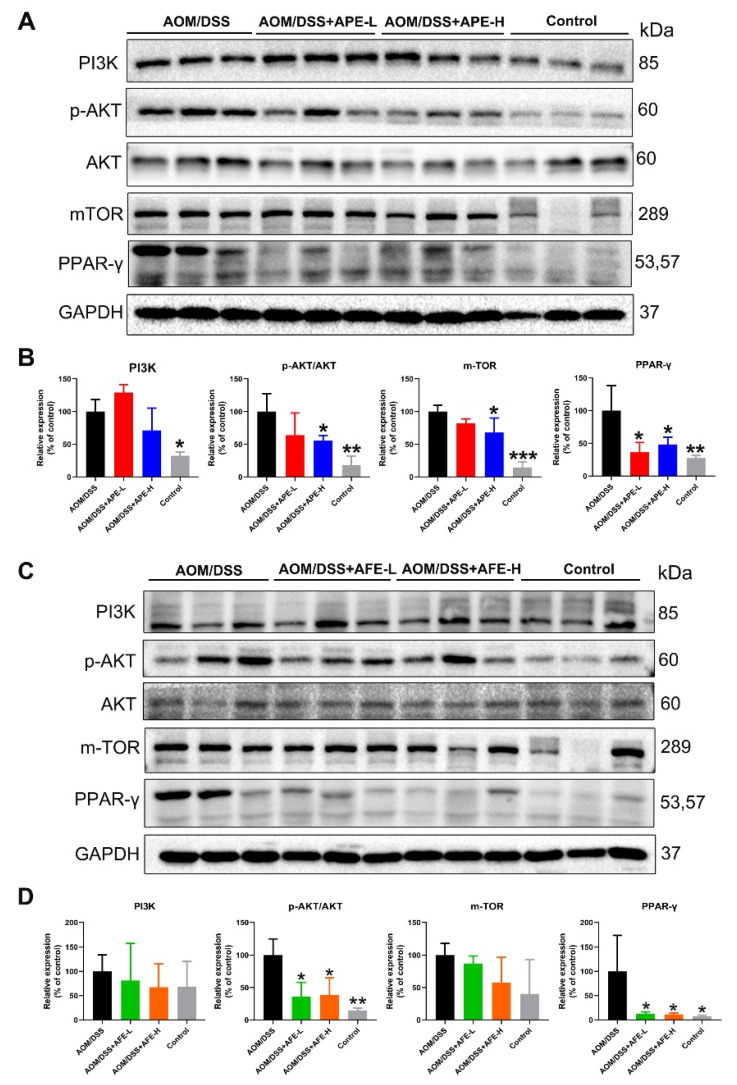
APE and AFE inhibited PI3K/AKT/mTOR signal transduction in the colonic tissues of the CAC mice. (**A**) APE suppressed the expression of PI3K/AKT/mTOR signal transduction. (**B**) The relative intensities of PI3K, p-AKT/AKT, m-TOR, and PPAR-γ expression after normalization to GAPDH. (**C**) AFE inhibited the expression of PI3K/AKT/mTOR signal transduction in the colonic tissues. (**D**) The relative intensities of PI3K, p-AKT/AKT, m-TOR, and PPAR-γ expression after normalization against GAPDH. Data are presented as the mean ± SD; * *p* < 0.05, ** *p* < 0.01, *** *p* < 0.001 vs. AOM/DSS group.

In our study on the AOM/DSS group, PI3K/AKT/mTOR signal transduction was activated and PPAR-γ expression was increased as compared with the control group (Figure 7A–D). However, the expressions of PI3K, p-AKT/AKT, mTOR, and PPAR-γ were significantly decreased with APE and AFE treatment in comparison with the model group (Figure 7A–D).

#### 3.6.2. APE and AFE Partly Regulated the Wnt/β-Catenin Signaling Pathway in the AOM/DSS Mice 

**Figure 8:**
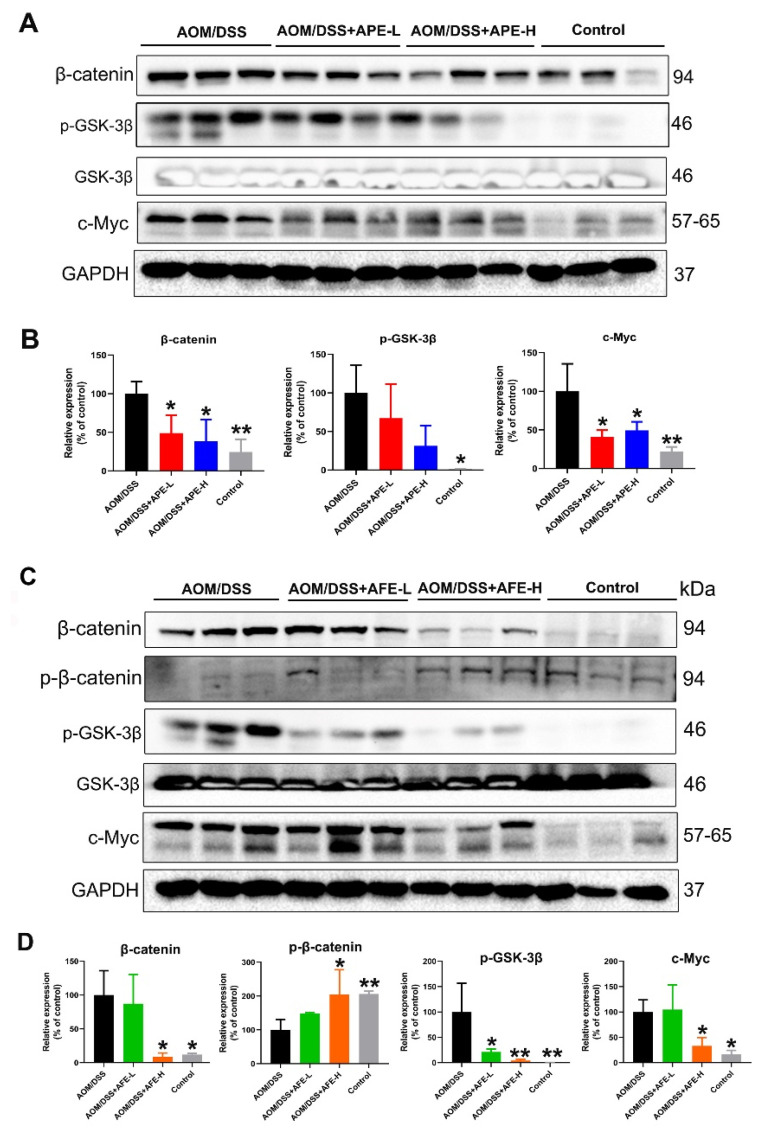
Effects of APE and AFE on Wnt/β-catenin signaling-related genes in the colonic tissues of the AOM/DSS mice. (**A**) APE inhibited the expression of Wnt/β-catenin signal transduction in colonic tissues. (**B**) The relative intensities of β-catenin, p-GSK-3β, and c-Myc after normalization against GAPDH. (**C**) AFE inhibited the expression of Wnt/β-catenin signal transduction in colonic tissues. (**D**) The relative intensities of β-catenin, p-β-catenin, p-GSK-3β, and c-Myc expression after normalization against GAPDH. Data are presented as the mean ± SD; * *p* < 0.05, ** *p* < 0.01 vs. AOM/DSS group.

In the present study, β-catenin, p-GSK-3β, and c-Myc, the downstream target genes of Wnt signaling, were overexpressed in the AOM/DSS group (Figure 8A–D), while APE and AFE were able to alleviate the overexpression of β-catenin, p-GSK-3β, and c-Myc in the colon of the AOM/DSS-treated mice in comparison with the model group. Moreover, AFE was able to increase the relative level of p-β-catenin. Together, these results imply that APE and AFE attenuate the development of CAC by suppressing the activation of the Wnt/β-catenin signaling pathway.

### 3.7. AFE Inhibited CT26 Colon Cancer Cell Growth and Metastasis through the Regulation of Multiple Pathways

**Figure 9:**
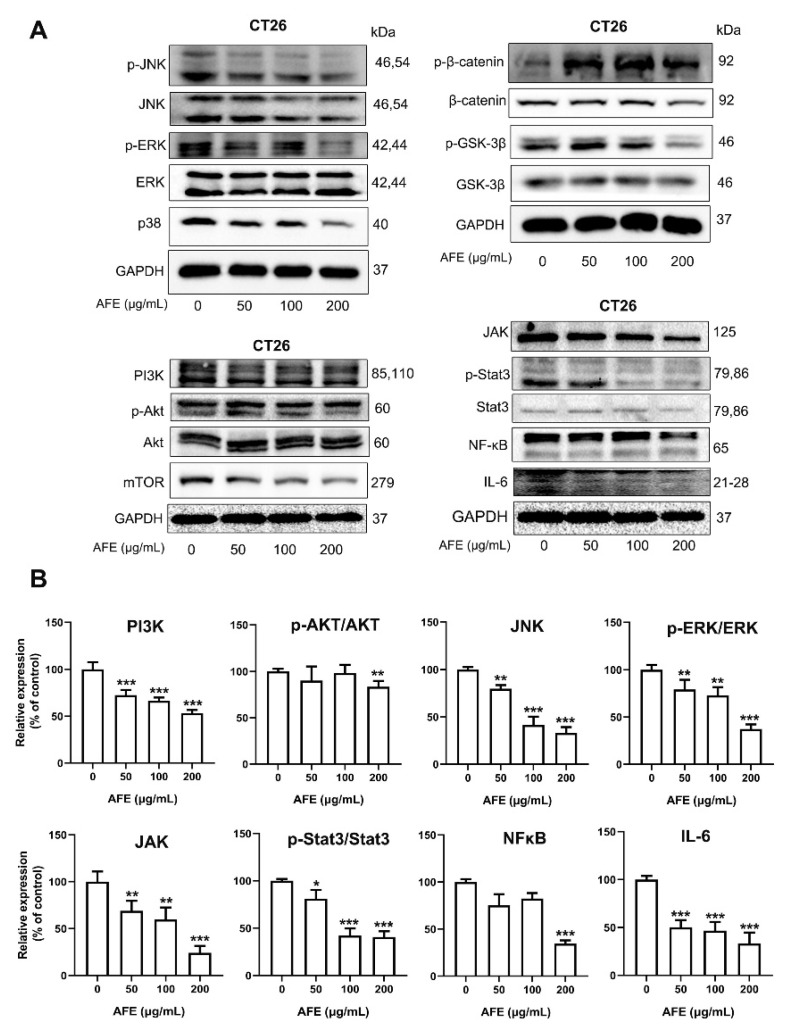
Effects of AFE on MAPK, Wnt/β-catenin, PI3K/AKT/mTOR, JAK2/Stat3, and NfκB/IL-6/Stat3 signaling transductions in CT26 cells. (**A**) Dose effects of AFE (0, 50, 100, and 200 μg/mL) on MAPK, Wnt/β-catenin, PI3K/AKT/mTOR, JAK2/Stat3, and NfκB/IL-6/Stat3 signaling transductions after treatment for 24 h in CT26 cells. (**B**) The relative intensities of the PI3K, p-AKT/AKT, JNK, p-ERK/ERK, JAK2, p-Stat3/Stat3, NFκB and IL-6 levels normalized against total proteins. Data are presented as the mean ± SD; * *p* < 0.05, ** *p* < 0.01, *** *p* < 0.001 vs. AOM/DSS group.

Similar to what we observed in vivo, AFE markedly reduced the relative expression of NFκB, IL-6, p-Stat3/Stat3, JAK2, JNK, p-ERK/ERK, PI3K, and P-AKT/AKT in the CT26 cells upon AFE treatment for 24 h (Figure 9A,B) in comparison to the control group. It turned out that AFE may elicit its anticancer activity through regulation of NFκB/IL-6/Stat3, JAK2/Stat3, MAPKs, PI3K/AKT, and Wnt/β-catenin signal transductions in CT26 cells. 

### 3.8. APE and AFE Treatment Modulated the Diversity and Composition of the Gut Microbiota 

**Figure 10:**
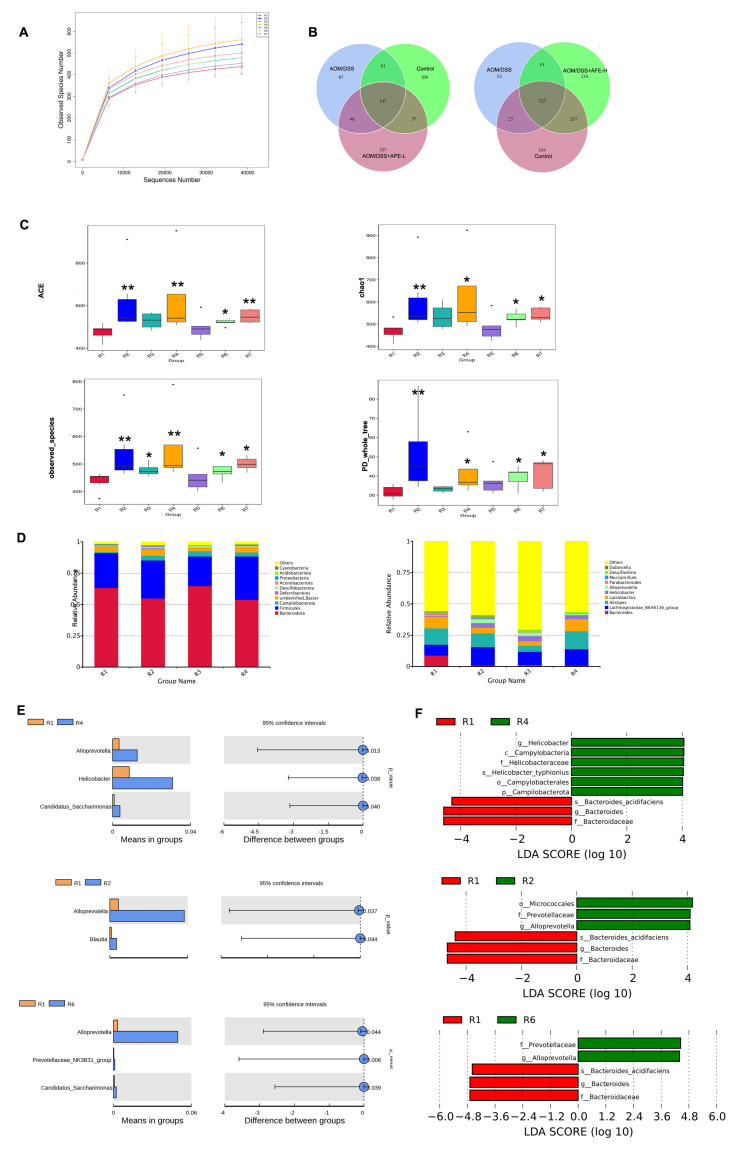
APE and AFE modulated the diversity and structure of the intestinal flora in the CAC mice. (**A**) Rarefaction curve. (**B**) The OTUs of each group. (**C**) Alpha diversity analyzed by ACE, Chao 1, observed_species, and PD_whole_tree. (**D**) The relative abundance of the flora at various levels. (**E**) T_test analysis of the different species between test groups. (**F**) LEfSe analysis of the dominant biomarker taxa. Data are presented as the mean ± SD; * *p* < 0.05, ** *p* < 0.01 vs. AOM/DSS group.

Intestinal dysbacteriosis has been observed in the CAC mouse model in numerous reports. We further tested whether APE/AFE treatment could ameliorate the intestinal dysbacteriosis caused by AOM/DSS in the C57BL/6 mice and conducted 16S rRNA sequencing of their feces. The terminal of the dilution curve of each group tended to be flat (Figure 10A), implying that the sample sequencing data were rational and that the vast majority of the flora diversity was caught. 

The Venn diagram (Figure 10B) shows that there were fewer OTUs in the AOM/DSS group than in the control group, but APE and AFE treatment increased the OTUs. The α-diversity analysis using ACE, Chao1, observed_species, and PD_whole_tree (Figure 10C) was evaluated, and these indices were markedly downregulated in the model group compared with the control group. After treatment with APE/AFE, these indices reverted to an equal level compared to the control group.

We detected changes in bacterial abundance at multiple levels via taxon analysis to assess the action of APE/AFE on microbiome structure remodeling. At the phylum level (Figure 10D), the most abundant *Firmicutes* and *Bacteroidetes* constituted over 84% of the total microbiota of all the samples. Evidently, in comparison with the control group, a higher abundance of *Bacteroidetes* and *Proteobacteria* and a lower abundance of *Firmicutes* were discovered in the AOM/DSS group. Nevertheless, the ratio of *Firmicutes* to *Bacteroidetes* (F/B) was elevated after intervention with AFE, with a decrease in *Proteobacteria*. Moreover, the relative abundance of *Campilobacterota* and *Actinobacteriota* was lower than that in the control group, but APE and AFE treatment increased their abundance. At the genus level (Figure 10E), the relative abundance of *Bacteroides*, *Parabacteroides*, *Parasutterella*, *Odoribacter*, and *Streptococcus* in the AOM/DSS group was elevated in comparison with those in the control group. Interestingly, APE/AFE administration entirely inhibited the increase in the relative abundance of the above bacteria caused by AOM/DSS. Furthermore, in comparison with the control group, lower abundance of *Lachnospiraceae_NK4A136_group*, *Oscillibacter*, *Alloprevotella,* and *Prevotella* was observed in the model group, and AFE and APE partially or even entirely reverse the decline in the relative abundance of above bacteria caused by AOM/DSS. 

A *t*-test was applied to make a distinction between the groups, which indicated that AOM/DSS treatment significantly decreased the relative abundance of *Alloprevotella* (*p* < 0.05), and APE/AFE treatment reversed the decrease (Figure 10F). The LEfSe analysis (LDA score: 4.0) of the present study showed that three taxa were influenced in the AOM/DSS group compared to the control group, which had six markedly changed taxa. When comparing the AOM/DSS group with the APE intervention group, there were three changed taxa in the model group and two in the APE intervention group. And when comparing the AOM/DSS group with the AFE intervention group, there were three taxa that were influenced by the AFE intervention, and three changed taxa in the AOM/DSS group.

Generally, these results further indicate that APE/AFE is critically involved in shaping the intestinal flora in the AOM/DSS-induced CAC mouse model.

## 4. Discussion

Okra, also known as qiukui, is an annual herb and mainly grown in immature pods. There exists massive flower waste during the fruit development period, which is a byproduct of okra but rich in polysaccharides and flavonoids. However, except for a few health teas, the development and utilization of okra flowers is limited. Epidemiological studies and experimental studies have linked CRC progression with dietary factors and identified that phytochemical-rich plant foods exert protective activities in CRC prevention [8]; dietary intervention has become an attractive strategy to inhibit its occurrence and progression. Owing to the high level and potential bioactivities of flavonoids and polysaccharides, okra flowers have potential to be novel agents. A previous study found that AFE exhibited anti-cancer activity in a CRC cell model and a CT26 xenograft mouse model [13]. And in this study, we applied the mutagenic agent AOM and the proinflammatory reagent DSS to induce CAC, mimicking the inflamed colon and carcinogenesis circumstances in humans, to investigate the effect of polysaccharide and flavonoid extracts from okra flowers on AOM/DSS-induced colitis and CAC, which can properly reflect the features and availability of agents in practical applications [21], which has never been reported before. 

We showed that APE and AFE treatment alleviated AOM/DSS-induced colitis, CAC, and CAC-caused dysbiosis. Preceding studies have reported that CRC is often accompanied by decreased diversity and richness of the gut microbiota, including in AOM/DSS-induced CRC models and CRC patients [22,23], and the structure of the microbiome decides the susceptibility to colorectal tumorigenesis [24]. Previously, okra flowers were known as a good source of flavonoids and polysaccharides, and mounting evidence has indicated a promising role of plant-derived polysaccharides and flavonoids as prebiotics that are able to provide protection against CRC by modulating the intestinal flora [25,26,27]. In this study, we proved that APE/AFE treatment elevated the richness and diversity of microbial composition compared to the AOM/DSS group. At the phylum level, *Firmicutes* was the most predominant phylum in all groups, exceeding 60%, which is important for butyrate production, has anti-inflammatory and anticarcinogenic effects, and has documented effects on CRC [28]. And Bacteroidetes, accounting for 10–15%, are significantly associated with tumor susceptibility and promotion and are involved in the development of CRC [29]. Previous studies reported that intestinal dysbacteriosis in the AOM/DSS group was visible at the phylum level, with a shift toward a decreased *Firmicutes*/*Bacteroidetes* ratio [29]. Consistent with previous studies, a decrease in *Firmicutes*/*Bacteroidetes* was observed in the model group in comparison with the control group, whereas it was improved through AFE treatment. In addition, AFE intervention elevated the relative abundance of *Proteobacteria* (decreased through AOM/DSS induction), and a lower abundance of which would be present in healthy humans but with an obvious preponderance in CAC [30]. The analysis at the genus level showed that *Bacteroides*, *Parasutterella*, and *Streptococcus*, which are positively associated with colorectal carcinogenesis, were increased by AOM/DSS treatment, whereas APE/AFE administration reversed the increase in these bacteria. In addition, APE/AFE intervention raised the relative abundance of the SCFA-producing bacterium Alloprevotella, which was diminished by AOM/DSS; it was identified to be an anti-inflammatory bacterium [31] and possesses the action of fermenting carbohydrates and producing SCFAs (acetate and butyrate) [32]. These results showed that APE/AFE treatment could attenuated CAC-caused dysbiosis by increasing the richness and diversity of microbial composition and modulating the structure of intestinal microflora, thus restraining CRC development. 

Chronic inflammation as well as the level of inflammatory mediators play critical roles in the initiation and development of CRC [33]. Macrophages always serve as essential effector cells in the maintenance of gut homeostasis and important sentinels of the gut immune system, which can induce intestinal inflammation through the proinflammatory cytokines expressed by the inflammatory response [34]. It was observed that APE/AFE treatment downregulated the expression of EMR1 (a macrophage cell marker) in the AOM/DSS-induced CAC mice, and EMR1 is a significant component of the leukocytic infiltration of tumors and has been perceived as a paradigm for cancer-related inflammation [14]. And APE/AFE treatment reduced the level of the inflammatory mediators COX-2 and IL-6 in the AOM/DSS-induced CAC mouse model; the two are often elevated in humans or in AOM/DSS-induced CRC mice. Continuous colonic proinflammatory macrophages releasing proinflammatory cytokines, such as IL-6, which is one of the most important proinflammatory cytokines connected with CRC, can aggravate colonic inflammation and tumorigenesis. 

Specifically, previous studies reported that IL-6 activated by NFκB could promote CAC progression via the NFκB/IL-6/Stat3 cascade; among them, NFκB is a significant regulator of the growth and survival of tumor-initiating IECs and following the release of IL-6 could induce the recruitment and subsequent phosphorylation of JAK2 and Stat3 [35]. The NF-κB/IL-6/Stat3 and JAK2/Stat3 pathways may be critically involved in CAC tumorigenesis, and it was found that AFE ameliorated colitis and inhibited CAC by downregulating these two inflammation-related signaling pathways. Unlike AFE, the results in the present study implied that APE treatment activated Nfr2 levels in the colonic tissue of the mice, which has become a novel target for CRC prevention recently, suggesting that the antioxidant capacity was significantly elevated through APE treatment, protecting against oxidative stress and inflammation due to AOM/DSS. 

The activation of MAPKs (including Erk, p38MAPK, and JNK), which has been proven to have a positive correlation with several inflammatory cytokines, including IL-6, is recognized as being able to interact with, expand, and integrate signals from various stimuli to regulate cellular proliferation, inflammatory responses, apoptosis, etc., and is critically involved in the development of IBD and CRC [36]. Furthermore, several accumulated inflammatory cytokines triggered by MAPKs signaling pathways could accelerate cell viability, proliferation, and metastasis via activating transcription factors, including NFkB and PPAR-γ [37,38,39]. In our study, AFE restrained the activation of JNK and p-ERK and downregulated the level of its downstream target gene c-fos, probably implying that AFE suppressed the inflammatory response, reduced colon injury, and impeded CRC development via NF-κB/IL-6/Stat3, JAK2/Stat3, and MAPK signal transductions. As for APE, it was found that APE suppressed the overexpression of JNK and p-ERK but increased P38 expression, which may lead to the activation of Nrf2 and inhibit the production of proinflammatory cytokines, exhibiting a protective effect on oxidative stress and inflammation to hamper colon tumorigenesis. 

The occurrence and development of CRC is a complex and multistage process involving the mutation of certain genes (APC, Kras, and P53) and the alteration of several signaling pathways (PI3K/AKT, Wnt/β-catenin, etc.), which are critically involved in the regulation of CRC cell growth, differentiation, angiogenesis, apoptosis, survival, and other biological processes. On the one hand, previous articles have reported that alterations in the PI3K pathway are universal in CRC, wherein activation of the PI3K pathway could contribute to the malignant transformation of benign lesions [40], and the overexpression of PI3K/Akt/mTOR signal transduction could exacerbate the progression of CRC [41]. In this research, we observed that APE/AFE treatment downregulated the PI3K level and the p-AKT/AKT ratio in the AOM/DSS-induced CAC mouse model and inhibited the expression of its downstream targets, including mTOR and PPAR-γ. Because of the prominent parts in the initiation and development events of cancer, many reports have indicated that inhibiting PI3K/AKT signals has been recognized as a potential therapeutic agent in CRC [42]. The results imply that APE/AFE administration inhibited CAC by inactivating PI3K/Akt/mTOR signal transduction and its downstream target PPAR-γ, which might be a latent agent for the blocking of CRC genesis and progenesis.

On the other hand, activated AKT could induce the phosphorylation of downstream targets, like GSK-3β, which induces β-catenin translocation to the nucleus, activation of the Wnt/β-catenin signal transduction, and the guidance of CRC development. In our study, a reduction in the phosphorylation of GSK-3β, which implies GSK-3β inactivation, without influencing total GSK-3β levels, was found after APE/AFE intervention, implying that APE/AFE abrogated Wnt/β-catenin signals by enhancing GSK-3β activity. The results are in accordance with the previous literature on anti-CRC natural components that inhibited Wnt/β-catenin signaling via activation of the Wnt destruction complex and promotion of β-catenin degradation [43]. Moreover, the expression of c-Myc, one of the critical components of Wnt/β-catenin signal target genes and the proto-oncogene encoding a transcription factor, was inhibited by APE/AFE administration [44]. 

Based on the these signaling pathways, APE and AFE could alleviate tumorigenesis and development in the AOM/DSS-induced CAC mice through regulating inflammatory signaling pathways and CRC-associated signaling pathways. In addition, we also found that AFE exhibited antioxidant activity by activating Nrf2 expression in CRC cells (Figure S1), but it elicited anticancer activity through regulation of PI3K/AKT, MAPK, Wnt/β-catenin, JAK2/Stat3, and NFκB/IL-6/Stat3 signal transductions in CT26 cells, which was consistent with what we observed in the AOM/DSS-induced CAC mice. While APE treatment had no obvious impact on the viability of CT26 and HCT116 cells in vitro, and APE showed weaker scavenging against ABTS and DPPH free radicals at a dose of 10 mg/mL, in comparison with Vc (10 μg/mL) (Figure S2). The above results revealed that AFE, flavonoids from okra flowers, both in vitro and in vivo, exhibited anti-CRC activity by regulating the same signaling pathways; however, APE, polysaccharides from okra flowers, inhibited AOM/DSS-induced colitis and tumorigenesis in mice without any effects on CRC cell viability or free radicals in vitro.

Compared to traditional chemotherapy agents, natural products have attracted much attention as candidates, as these natural products interfere with a wide variety of signaling pathways; meanwhile, they are regarded as multitargeted. In this report, we extracted the water-soluble crude polysaccharides APE and ethanol-soluble flavonoids AFE both from okra flowers, which allowed us to explore the functional capacity of two different bioactivities from one plant. It provides expansion to the field of CRC prevention and treatment, and is also a driving force for the development of studies on okra flowers. 

## 5. Conclusions

In our study, we applied AOM/DSS to duplicate a mouse model of CRC to examine the anti-CRC activity of APE and AFE from okra flowers. APE and AFE treatment alleviated AOM/DSS-induced colitis, CAC, and CAC-caused dysbiosis in the mice, accompanied by enhancing gut barrier function and regulating inflammatory signaling pathways and CRC-associated signaling pathways. Collectively, the present study designated APE/AFE as a promising agent in alleviating IBD and CAC. But more detailed mechanisms of APE/AFE in attenuating IBD and CAC should be explored in the future, such as whether the functional capacity of APE/AFE is mediated by gut microbiota manipulation and how exactly the altered gut microbiota regulated by APE/AFE intervention affects the CRC progression.

## Figures and Tables

**Table 1:** The disease activity index score of the mice.

Score	Weight Loss (%)	Stool Consistency	Visible Fecal Blood
0	<1	Normal pellets	Normal
1	1–5	Loose feces	Slightly bloody
2	6–10		
3	11–15		
4	>15	Watery diarrhea	Blood in the entire colon

## Data Availability

Data are contained within the article and Supplementary Materials.

## Supplementary Materials

The following supporting information can be downloaded at: https://www.mdpi.com/article/10.3390/nu15224820/s1, Figure S1: AFE exerted antioxidant activity in CRC cells through activation of Nrf2 expression. Figure S2: The extraction method of APE, and its effect on cell activity of CRC cancer and free radicals (DPPH and ABTS).
