# Supplementary figures and images for: Chemopreventive Effects of Polysaccharides and Flavonoids from Okra Flowers in Azomethane/Dextran Sulfate Sodium-Induced Murine Colitis-Associated Cancer
Source: Nutrients. 2023 Nov 17;15(22):4820. doi: 10.3390/nu15224820 (PMC10674164; doi:10.3390/nu15224820)

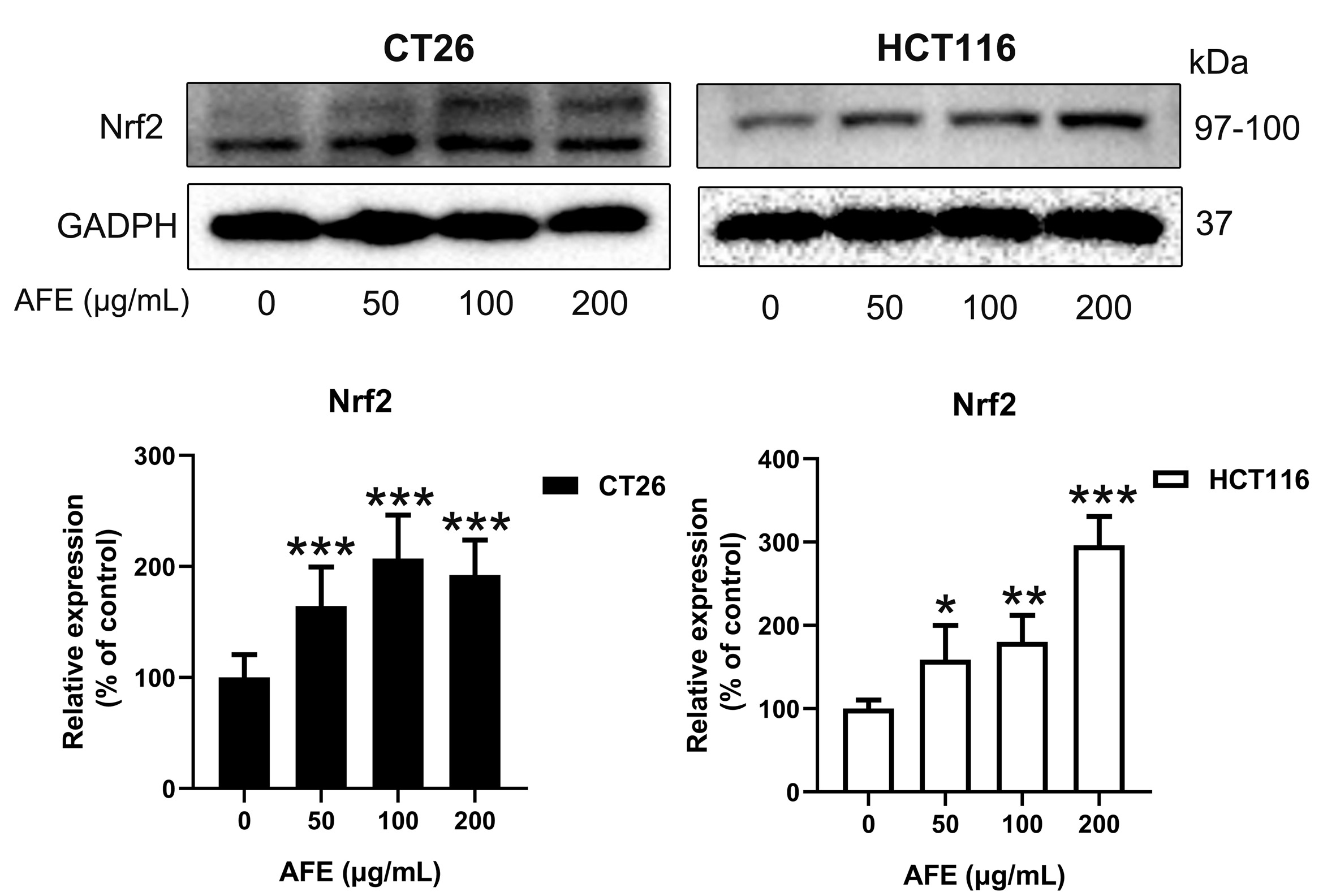

Supplement: Supplementary file 1 [file nutrients-15-04820-s001.zip › nutrients-2683092-supplementary/Figure S1.jpg]

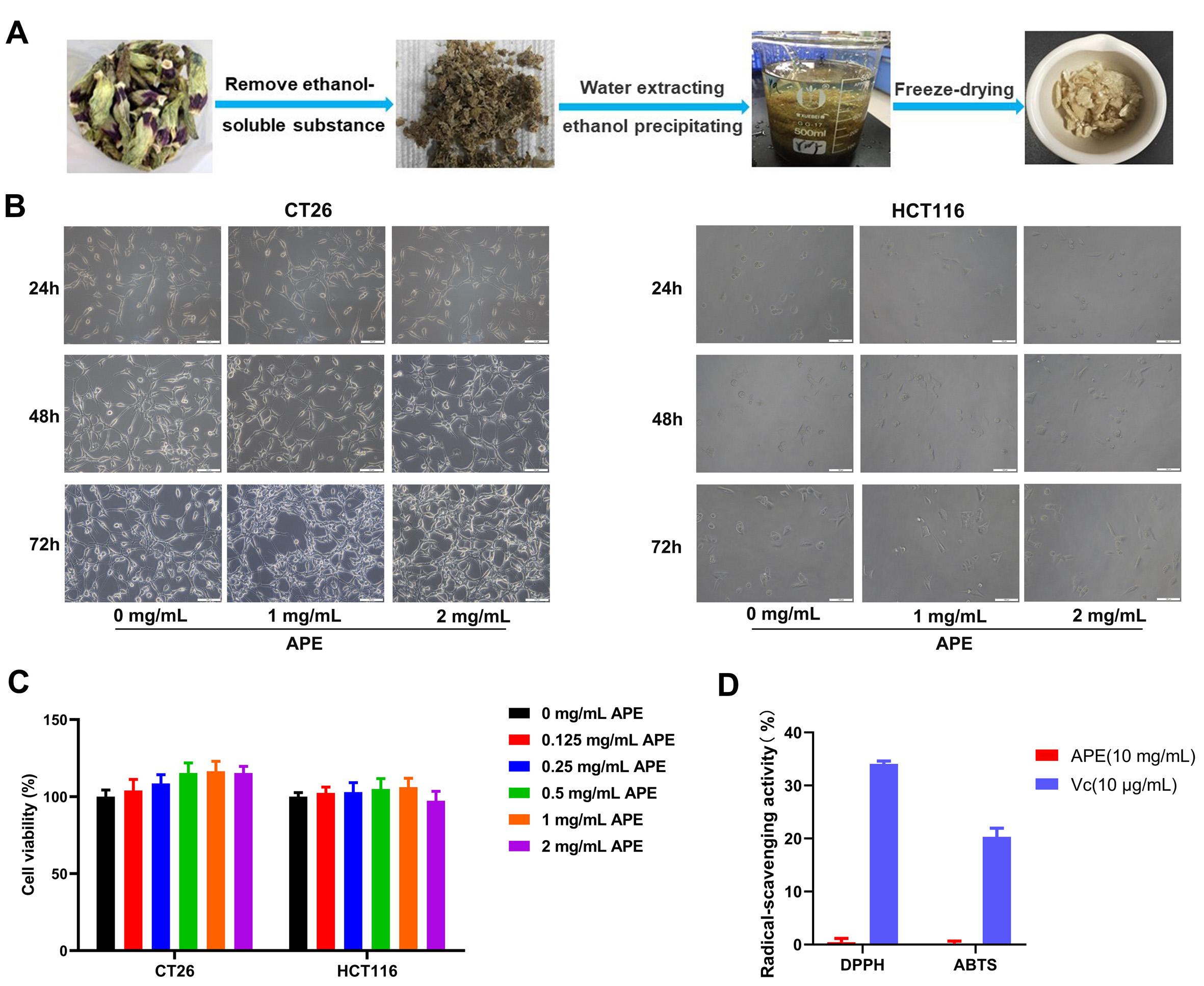

Supplement: Supplementary file 1 [file nutrients-15-04820-s001.zip › nutrients-2683092-supplementary/Figure S2.jpg]
